# Factors influencing the transition phase in acute respiratory distress syndrome: an observational cohort study

**DOI:** 10.1186/s13613-025-01484-6

**Published:** 2025-05-26

**Authors:** Anne-Fleur Haudebourg, Louise Chantelot, Safaa Nemlaghi, Luc Haudebourg, Pascale Labedade, Mohamed Ahmed Boujelben, Guillaume Voiriot, Armand Mekontso Dessap, Muriel Fartoukh, Guillaume Carteaux

**Affiliations:** 1 Service de Médecine Intensive Réanimation, Assistance Publique-Hôpitaux de Paris, CHU Henri Mondor-Albert Chenevier, 51, Avenue du Maréchal de Lattre de Tassigny, Créteil Cedex, 94010 France; 2 Faculté de Santé, Groupe de Recherche Clinique CARMAS, Université Paris Est-Créteil, Créteil, 94010 France; 3 INSERM U955, Institut Mondor de Recherche Biomédicale, Créteil, F-94010 France; 4 Service de Réanimation médicale et infectieuse, Assistance Publique-Hôpitaux de Paris, Hôpital Bichat-Claude Bernard, 46, rue Henri Huchard, Paris, 75018 France; 5 Service de Médecine Intensive Réanimation, Assistance Publique-Hôpitaux de Paris, Hôpital Tenon, 4, rue de la Chine, Paris, 75020 France; 6 Service de Médecine Intensive Réanimation, CHU Henri Mondor, 1, rue Gustave Eiffel, Créteil Cedex, 94010 France

**Keywords:** Mechanical ventilation, Respiratory mechanics, Acute respiratory distress syndrome, Protective ventilation

## Abstract

**Background:**

Protective ventilation during the acute phase of ARDS and weaning from mechanical ventilation are well-established in current guidelines. However, the intermediate transition phase between these stages remains poorly characterized.

**Objectives:**

To describe the transition phase in moderate-to-severe ARDS and evaluate the factors associated with neuromuscular blockade (NMBA) weaning failure and pressure support ventilation (PSV) failure.

**Methods:**

This bicentric observational cohort study included patients with moderate-to-severe ARDS requiring NMBA continuous infusion within 72 h post-intubation. The transition phase was defined as the 72 h following the first NMBA weaning attempt. The main endpoints were the rates of NMBA reintroduction and PSV failure. Secondary outcomes included predictive factors for NMBA weaning failure and PSV failure and the impact of tidal volume on patient outcomes.

**Main results:**

A total of 196 patients were included. NMBA weaning failure occurred in 74 (38%) patients. COVID-19 (OR 3.98 [1.95–8.41], *p* < 0.001), pH (OR 0.50 [0.30–0.79], *p* = 0.004), PaO_2_/FiO_2_ ratio (OR 0.92 [0.87–0.97], *p* = 0.007), and high or low driving pressure before first NMBA weaning attempt (< 12 or ≥ 14 cmH_2_O) (OR 2.77 [1.16–7.14], *p* = 0.027) were significantly associated with NMBA reintroduction. PSV was initiated in 147 (75%) patients, with a failure rate of 57%, occurring after a median of 9 h [6–24]. Tidal volume (OR 1.28 [1.06–1.56], *p* = 0.012) was significantly associated with PSV failure. During PSV, 43% of patients exhibited high tidal volumes (> 8 mL/kg PBW). NMBA weaning failure was associated with fewer ventilator-free days and increased mortality at day 28. PSV failure was associated with fewer ventilator-free days.

**Conclusion:**

The transition phase represents a high-risk period in ARDS, with significant failure rates for NMBA weaning and PSV trials that may influence patient outcomes. The transition phase therefore represents a critical area for future research to optimize management during this vulnerable period.

**Supplementary Information:**

The online version contains supplementary material available at 10.1186/s13613-025-01484-6.

## Background

Acute respiratory distress syndrome (ARDS) accounts for 10.4% of ICU admissions and is associated with a mortality rate of approximately 40% [1]. Ventilation strategies during the early phase of ARDS have been extensively studied, leading to updated recommendations [2–4]. During this phase, the use of low tidal volume remains the cornerstone of protective ventilation [5]. Current guidelines also recommend the use of neuromuscular blocking agents (NMBA) for up to 48 h in patients with moderate-to-severe ARDS [2, 3], even though their use has been debated following the ROSE study [6], which failed to replicate the benefit of decreased mortality observed in the ACURASYS trial [7]. While there is a growing trend toward limiting NMBA duration in patients showing clinical improvement [3], clearly defined discontinuation criteria are lacking, leading to uneven practices [8]. Closer to the end of the mechanical ventilation course, weaning from mechanical ventilation has also been widely investigated and is the subject of recommendations [9, 10]. In contrast, the period between the acute phase of protective ventilation and the weaning phase, referred to here as the transition phase, remains poorly described in the literature. This intermediate phase may be critical, as it involves key steps for patients who have not fully recovered, such as NMBA weaning, the restauration of spontaneous breathing, and the transition from assist-control ventilation (ACV) to pressure support ventilation (PSV). The success rates of these steps and the potential consequences of their failure remain unknown. During this phase, excessive respiratory effort can lead to harmful physiological effects, including pendelluft [11], uncontrolled tidal volumes, myotrauma [12–14], and the aggravation of pulmonary edema through hemodynamic mechanisms. Experimental studies have demonstrated exacerbated lung injury associated with higher respiratory effort [15, 16], supporting the concept of patient self-inflected lung injury (P-SILI) [17]. A notable concern is the potential role of high tidal volumes resulting from excessive respiratory effort during the transition phase, as their impact remains unknown. In non-invasive ventilation (NIV) for *de novo* acute hypoxemic respiratory failure, high tidal volumes have been identified as independent risk factors for NIV failure and mortality [18–20].

The objective of this study was to describe the transition phase in patients with moderate-to-severe ARDS, defined as the 72 h following the first NMBA weaning attempt, and to identify factors associated with the failures of NMBA weaning attempt and assisted ventilation trial. We also aimed to explore the potential association between tidal volume under PSV during the transition phase and patient outcomes.

## Methods

### Study design

We conducted a retrospective observational cohort study in two tertiary intensive care units (ICU) during two periods: the first from 2014 to 2017, involving ARDS from all causes, and the second from March to June 2020, focusing on Covid ARDS. Patients were screened for the study if they met the criteria for moderate-to-severe ARDS according to the Berlin definition [21] and had received neuromuscular blockade for at least 12 h within the first 72 h post-intubation. They were considered to fulfill the inclusion criteria at the first attempt by the treating physician to withdraw NMBA (marking the entrance into the transition phase; see below). Non-inclusion criteria were the following: age < 18 years, pregnancy or breastfeeding, extra-corporeal membrane oxygenation, severe central neurological impairment, cardiac arrest, diaphragmatic paralysis, a decision to withhold life-sustaining treatment, death prior to entry into the transition phase, and patients under legal protection. The study was approved by the ethics committee of the Société de Réanimation de Langue Française (French Society of Intensive Care Medicine; IRB 00014135).

### Transition phase definition

The transition phase was defined as the 72-hour period following the first withdrawal of NMBA, a duration arbitrarily chosen for the purposes of this study.

### Data collection

We collected the following data from patient records:

- From admission to transition phase entry: demographic data, comorbidities, ARDS etiology, severity scores (SOFA, SAPS II), sedation and NMBA dosages, use of prone positioning, nitric oxide, renal replacement therapy, and vasopressor use, as well as ventilatory parameters (ventilatory modes, tidal volume, respiratory mechanics) and arterial blood gases;
- During transition phase: ventilatory parameters and NMBA usage were recorded every three hours. For patients on PSV, the mean values for ventilatory variables were calculated as the average of the data collected at three-hour intervals. Arterial blood gases and SOFA scores were recorded daily;
- Follow-up until day 90: ICU and in-hospital mortality, mortality at day 28 and day 90, ICU length of stay (ICU-LOS), days of mechanical ventilation, and ventilator-free days (VFD) at day 28.

### Endpoints

The primary objectives were to identify the rate of neuromuscular blockade (NMBA) weaning failure and to determine the predictive factors associated with this failure, as well as to identify the rate of pressure support ventilation (PSV) failure and its predictive factors. NMBA weaning failure was defined as the early resumption of continuous neuromuscular blockade within 48 h of the initial withdrawal. PSV failure was defined as the proportion of patients who were transitioned back to assist-control ventilation (ACV) during the 72-hour transition phase.

Secondary objectives included evaluating the impact on patient outcomes of NMBA weaning failure, PSV failure, and tidal volume observed during the transition phase.

### Statistics

All data were collected retrospectively from electronic patients’ records and verified by two independent reviewers. Continuous variables were expressed as medians with interquartile ranges (IQRs) or means with standard deviation (SD) as appropriate, while discrete variables were expressed as counts and percentages. Univariate analyses were performed using the Student’s t-test for continuous variables and the Chi-square test for discrete variables.

Associations between covariates and the outcomes of interest (i.e., neuromuscular blockade weaning failure at 48 h and PSV failure) were estimated using logistic regression models, and results are expressed as odds ratios (ORs) along with their 95% confidence interval (95% CI). Associations between covariates and VFD at day 28 were analyzed using Fine and Gray models to account for the competitive risk of death, with results expressed as hazard ratios (HRs) along with their 95% CI. Initial identification of covariates was performed using simple logistic regression models, with the effect of each covariate estimated through univariate analyses. Multivariate models were adjusted for potential confounders that were significantly associated with the outcome in univariate analyses, with a *p* value threshold of 0.05 or less. Among related factors, only the most clinically relevant were entered into the multivariable model to minimize the effects of collinearity. Receiver operating characteristic (ROC) curve analysis was used to evaluate the ability of pH, PaO_2_ on FiO_2_ ratio and tidal volume to predict NMBW weaning or PSV failure. Thresholds were determined using the Youden index to identify the optimal cutoff points.

All statistical analysis was performed using R Statistical Software (version 4.3.2, R Foundation for Statistical Computing, Vienna, Austria). All tests were two-sided, and *p*-values < 0.05 were considered statistically significant.

## Results

**Fig. 1:**
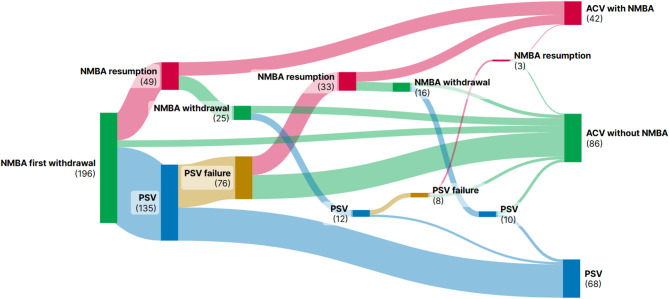
Sankey diagram displaying the course of the 196 patients during the 72-hour transition phase. The green box on the left size represents the first withdrawal of NMBA (i.e. the entry into the transition phase). The three boxes on the right size represent ventilatory mode of the patients at the end of the 72-hour transition phase (red box: assist-control ventilation with continuous NMBA infusion; green box: assist-control ventilation without continuous NMBA infusion; blue box: pressure support ventilation). The red boxes represent patients who need resumption of continuous neuromuscular blockade. The green boxes represent all subsequent attempts of NMBA withdrawal. The blue boxes represent patients who were transitioned to pressure support ventilation. The yellow boxes represent patients who were transitioned back to assist-control ventilation. Lanes show the status evolution of patient until they presented the different events during transition phase *Abbreviations*: NMBA: neuromuscular blocking agent; ACV: Assist-control ventilation; PSV: pressure support ventilation

A total of 196 patients were included in the study: 109 with non-COVID-19 ARDS (2014–2017) and 87 with COVID-19 ARDS (March-June 2020). There were less than 1% missing data, except for plateau pressure at the time of NMBA withdrawal, for which the missing data rate was 13% (*n* = 25). The main characteristics of the patients are summarized in Table 1. Patients spent a median of 2 days [1–5] under mechanical ventilation before entering the transition phase. The course of patients during the 72-hour transition phase is shown in Fig. 1.

**Table 1:** Characteristics of the population

	All patients *n* = 196	NMBA weaning failure *n* = 74	NMBA weaning success *n* = 122	*p*-value
**Demographic characteristics**				
Age, years	61 [52–69]	62 [53–69]	61 [51–68]	0.388
Sex, male, *n* (%)	155 (79.1)	59 (79.7)	96 (78.7)	1.000
BMI, kg/m^2^	27.5 [23.4–33.0]	27.8 [24.0–33.4]	27.3 [23.1–32.1]	0.172
Comorbidities:				
Chronic heart failure, *n* (%) Chronic kidney failure, *n* (%) COPD, *n* (%) Immunosuppression, *n* (%) Malignancy, *n* (%)	13 (7) 30 (15) 14 (7) 38 (19) 30 (15)	3 (4) 13 (18) 5 (7) 14 (19) 11 (15)	10 (8) 17 (14) 9 (7) 24 (20) 19 (16)	0.404 0.631 1.000 1.000 1.000
Time from ICU admission, days	3 [2–6]	3 [2–5]	3 [2–7]	0.957
Time from MV onset, days	2 [1–5]	2 [1–5]	2 [1–5]	0.291
Time from NMBA introduction, days	2 [1–4]	2 [1–4]	2 [1–4]	0.232
SAPS II at ICU admission	38 [29–46]	38 [31–47]	37 [28–46]	0.448
SOFA at ICU admission	7 [4–8]	6 [4–8]	7 [5–9]	0.409
SOFA at NMBA weaning	6 [4–9]	7 [5–10]	6 [4–8]	0.308
**ARDS cause**				
Pulmonary ARDS, *n* (%) *Including Covid-19*	150 (76.5) *87 (44.4)*	56 (75.7) *45 (60.8)*	94 (77.0) *42 (34.4)*	0.963 *0.001*
Extra-pulmonary ARDS, *n* (%)	46 (23.5)	18 (24.3)	28 (23.0)	
**Ventilatory parameters at ARDS onset (day 1 of invasive ventilation)**				
FiO_2_, %	100 [80–100]	100 [86–100]	100 [80–100]	0.242
Tidal volume, mL/kg PBW	6.1 [5.8–6.5]	6.1 [5.8–6.4]	6.1 [5.8–6.5]	0.715
Respiratory rate, breaths/min	26 [24–30]	26 [24–30]	26 [24–28]	0.155
PEEP, cmH_2_O	10 [6–12]	10 [8–12]	8 [5–10]	< 0.001
Driving pressure, cmH_2_O	13 [11–17]	12 [11–16]	14 [11–17]	0.388
Crs, mL/cmH_2_O	31 [25–38]	31 [26–39]	30 [23–37]	0.248
PaO_2_ on FiO_2_ ratio	116 [87–167]	119 [89–153]	114 [84–175]	0.423
Ventilatory ratio	1.79 [1.52–2.13]	1.86 [1.53–2.25]	1.77 [1.51–2.05]	0.211
**Treatments before NMBA weaning**				
Number of sedative drugs, *n* (%)				0.003
1 drug 2 drugs 3 drugs	142 (72.8) 48 (24.6) 5 (2.6)	56 (76.7) 12 (16.4) 5 (6.8)	86 (70.5) 36 (29.5) 0 (0.0)	
*Including ketamine*	*7 (3.6)*	*6 (8.1)*	*1 (0.8)*	*0.023*
NMBAs				
Atracurium, *n* (%)	195 (99.5)	74 (100.0)	121 (99.2)	
*Cumulative dose*, *mg*	*2075 [1088–5166]*	*2425 [1105–5246]*	*1950 [1090–5015]*	*0.162*
Vasopressors, *n* (%)	150 (76.9)	57 (77.0)	93 (76.9)	1.000
Nitric oxide, *n* (%)	13 (6.6)	7 (9.5)	6 (4.9)	0.346
Prone position, *n* (%) *Number of sessions*	100 (51.0) *1 [0–1]*	43 (58.1) *1 [0–2]*	57 (46.7) *0 [0–1]*	0.162 *0.097*
Renal replacement therapy, *n* (%)	23 (11.7)	9 (12.2)	14 (11.5)	1.000

*Abbreviations*: NMBA: neuromuscular blocking agent; BMI: body mass index; ICU: intensive care unit, MV: mechanical ventilation; SAPS II: simplified acute physiology score II; SOFA: sepsis-related Organ Failure Assessment; ARDS: acute respiratory distress syndrome; Crs: compliance of the respiratory system

### NMBA weaning

Ventilatory parameters and respiratory mechanics at the time of the first NMBA weaning attempt are presented in Table 2. At this stage, all patients were on volume-controlled ventilation, with a median tidal volume of 6.1 mL/kg PBW [5.8–6.5] and a median PEEP of 10 cmH_2_O [8–12]. Following the initial NMBA withdrawal, 74 patients (38%) required reintroduction of NMBA within the next 48 h for asynchronies or signs of respiratory distress despite deep sedation (*n* = 24; 32%), for isolated oxygenation impairment (*n* = 15; 20%), or prone positioning facilitation (*n* = 9; 12%). No specific reason for NMBA reintroduction was documented in the remaining 26 patients (35%). At the time of NMBA withdrawal, the majority of patients (*n* = 150; 78%) had improving ARDS compared to the post-intubation PaO_2_/FiO_2_ ratio, with no difference between the failure and the success group (59 patients (80%) and 91 (77%) respectively, *p* = 0.834). Conversely, 12 patients (16%) had worsening ARDS compared to intubation in the failure group vs. 23 (20%) in the success group (*p* = 0.834).

**Table 2:** Ventilatory parameters and respiratory mechanics at the onset of the transition phase (first withdrawal of neuromuscular blockers)

	All patients *n* = 196	NMBA weaning failure *n* = 74	NMBA weaning success *n* = 122	*p*-value
**Ventilatory parameters**				
FiO_2_, %	50 [40–60]	50 [50–64]	50 [40–60]	0.175
Tidal volume, mL	417 [371–450]	422 [383–456]	410 [366–448]	0.107
Tidal volume, mL/kg PBW	6.1 [5.8–6.5]	6.2 [5.9–6.5]	6.0 [5.7–6.4]	0.119
Respiratory rate, breaths/min	28 [25–32]	30 [26–32]	28 [25–32]	0.173
Minute ventilation, L/min	11.4 [10.0–13.2]	11.7 [10.3–13.9]	11.2 [9.9–12.7]	0.056
Peak pressure, cmH_2_O	37 [33–41]	38 [34–42]	37 [32–41]	0.257
Plateau pressure, cmH_2_O	24 [21–27]	24 [21–27]	24 [21–26]	0.348
PEEP, cmH_2_O	10 [8–12]	10 [10–13]	10 [8–12]	0.175
Driving pressure, cmH_2_O	12 [10–15]	12 [10–15]	12 [10–14]	0.927
*< 12 cmH*_*2*_*O*, *n* *(%)* *12–13 cmH*_*2*_*O*, *n* *(%)* *≥ 14 cmH*_*2*_*O*, *n* *(%)*	*70 (41)* *41 (24)* *59 (35)*	*29 (45)* *10 (17)* *25 (39)*	*41 (39)* *31 (29)* *34 (32)*	*0.047*
Crs, mL/cmH_2_O	34 [27–42]	34 [26–43]	33 [27–39]	0.686
Rrs, cmH_2_O/L/s	13 [10–16]	13 [10–16]	13 [10–16]	0.918
Mechanical power, J/min	26.3 [21.7–33.9]	27.6 [22.9–36.0]	25.9 [19.9–33.0]	0.032
4∆*P* + RR	78 [67–88]	79 [66–90]	78 [68–86]	0.615
**Blood gases**				
pH	7.40 [7.34–7.44]	7.37 [7.31–7.42]	7.40 [7.35–7.45]	0.001
PaCO_2_, mmHg	43 [38–48]	43 [39–50]	42 [38–48]	0.029
PaO_2_, mmHg	98 [79–132]	100 [77–128]	98 [79–135]	0.128
PaO_2_ on FiO_2_ ratio	196 [162–246]	190 [155–218]	198 [168–269]	0.005
Bicarbonates, mmol/L	26.9 [23.5–30.0]	25.9 [22.1–30.0]	27.0 [23.9–30.0]	0.526
Lactates, mmol/L	1.6 [1.2–2.1]	1.6 [1.3–2.0]	1.6 [1.1–2.2]	0.875
Ventilatory ratio	2.02 [1.69–2.31]	2.11 [1.80–2.48]	1.88 [1.60–2.25]	0.018

*Abbreviations*: NMBA: neuromuscular blocking agent; PEEP: positive end-expiratory pressure; Crs: compliance of the respiratory system; Rrs: resistance of the respiratory system

**Fig. 2:**
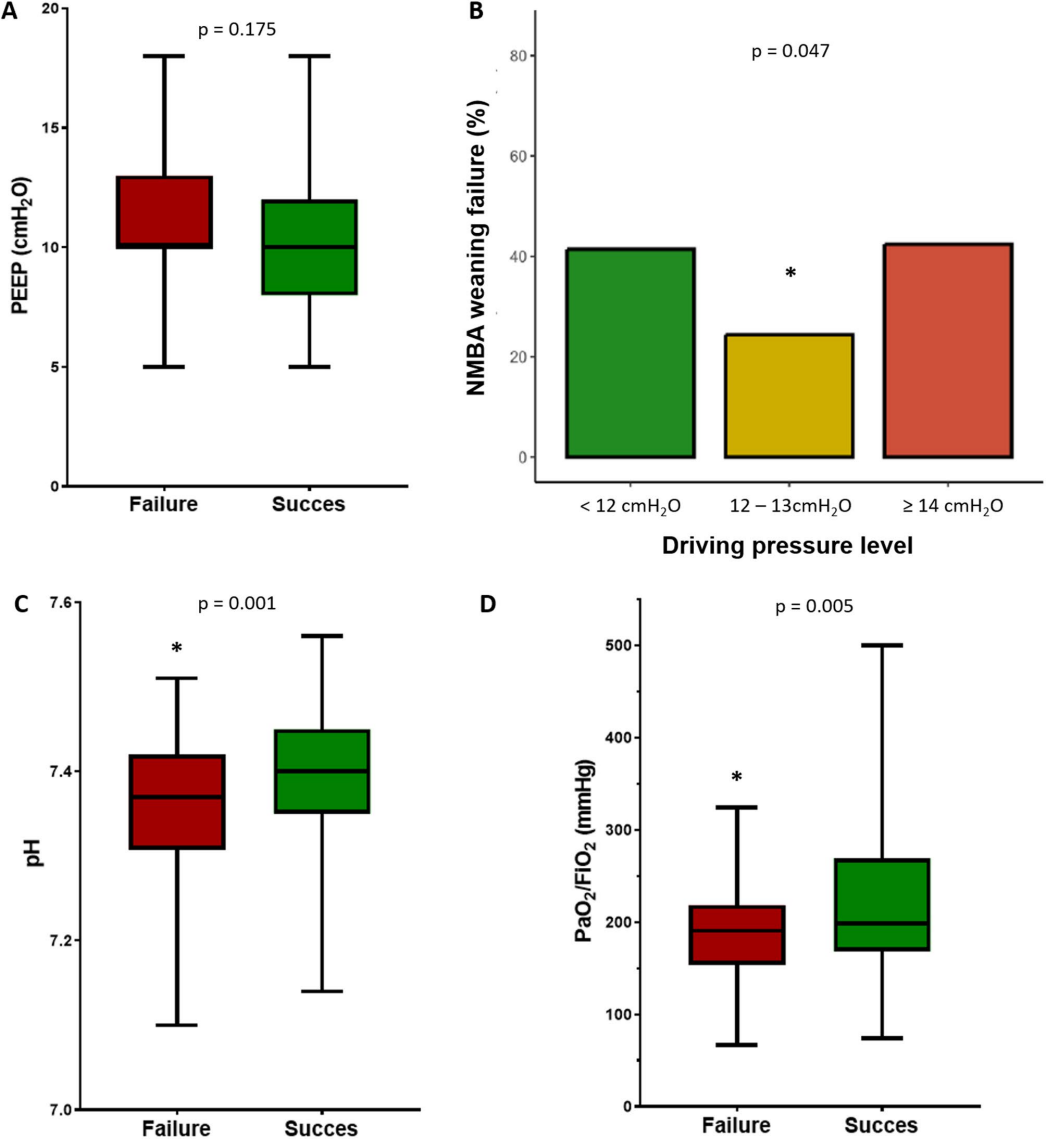
Risk factors for neuromuscular blocking agent weaning failure. **A**: Box-plot representing PEEP according to NMBA weaning failure or success (box: IQR 25–75; horizontal line within the box: median; whiskers: min and max values). **B**: Bar-plot showing the proportion of patients with NMBA reintroduction at 48 h of first NMBA withdrawal according to driving pressure level. **C**: Box-plot representing pH according to NMBA weaning failure or success (box: IQR 25–75; horizontal line within the box: median; whiskers: min and max values). **D**: Box-plot representing PaO_2_ on FiO_2_ ratio according to NMBA weaning failure or success (box: IQR 25–75; horizontal line within the box: median; whiskers: min and max values) * Denotes statistical significance *Abbreviations*: NMBA: neuromuscular blocking agent; PEEP: positive end-expiratory pressure

Univariate analysis identified several factors significantly associated with NMBA weaning failure, including COVID-19 as the cause of ARDS, driving pressure < 12 cmH_2_O or ≥ 14 cmH_2_O, mechanical power, pH, PaCO_2_, PaO_2_/FiO_2_ ratio and the ventilatory ratio at the time of NMBA withdrawal (Table 3). The following factors were independently associated with NMBA weaning failure by multivariate analysis: COVID-19 as the cause of ARDS, driving pressure < 12 cmH_2_O or ≥ 14 cmH_2_O, mechanical power, pH and PaO_2_/FiO_2_ ratio (Table 3; Fig. 2). Although significant association in univariate analysis, the ventilatory ratio was not include in the multivariate analysis due to its high collinearity with pH (see Table 3). However, when the ventilatory ratio is included in the model, it is not independently associated with NMBA weaning failure.

**Table 3:** Multivariate analysis of risk factors for neuromuscular blocking agent weaning failure

	Univariate			Multivariate		
	OR	95% IC	*p*-value	OR	95% IC	*p*-value
COVID-19	2.96	1.64–5.42	< 0.001	3.98	1.95–8.41	< 0.001
SOFA at NMBA weaning	1.05	0.96–1.14	0.307	-		
**Ventilatory parameters at NMBA weaning**						
Tidal volume, mL/kg PBW	1.45	0.91–2.34	0.12	-		
Respiratory rate, breaths/min	1.04	0.98–1.11	0.173	-		
PEEP, cmH_2_O	1.07	0.97–1.18	0.175	-		
Driving pressure (< 12 or ≥ 14 cmH_2_O)	2.23	1.04–5.15	0.047	2.77	1.16–7.14	0.027
Mechanical power, J/min	1.04	1.00–1.08	0.034	1.05	1.01–1.09	0.030
**Blood gases at NMBA weaning**						
pH	0.52	0.35–0.77	0.001	0.50	0.30–0.79	0.004
PaO_2_, mmHg	1.00	0.99–1.00	0.135	-		
PaCO_2_, mmHg	1.04	1.00–1.08	0.032	-		
PaO_2_/FiO_2_, mmHg	0.94	0.89–0.98	0.007	0.92	0.87–0.97	0.007
Ventilatory ratio	1.06	1.01–1.12	0.021	-		

*Abbreviations*: SOFA: sepsis-related Organ Failure Assessment; NMBA: neuromuscular blocking agent; PBW: predicted body weight; PEEP: positive end-expiratory pressure

Using receiver-operating characteristic (ROC) curves analysis, the accuracy of PaO_2_/FiO_2_ and pH in predicting NMBA weaning failure was limited, with area under the curve (AUC) values of 0.630 and 0.585, respectively. We therefore identified thresholds with high specificity (> 80%), which may help identify patients at high risk of NMBA withdrawal failure. A PaO2/FiO2 threshold of 150 mmHg and pH threshold of 7.30 yielded a specificity of 84% and 87% with a sensitivity of 23% and 24%, respectively. (Supplemental Material, Figure E1).

### Pressure support ventilation

During the transition phase, 147 patients (75%) were switched to PSV. Among them, 63 patients (43%) remained on PSV, while 84 (57%) were switched back to ACV after a median duration of 9 h [6–24] (Table 4). Among the remaining 49 patients who successfully underwent NMBA withdrawal but did not transition to PSV, 47 remained on volume-controlled ventilation throughout the entire transition phase, and 2 were switched to APRV after NMBA withdrawal.

**Table 4:** Patient’s characteristics during pressure support ventilation

	All patients *n* = 147	PSV failure *n* = 84	PSV success *n* = 63	*p*-value
**ARDS cause**				
Pulmonary ARDS, *n* (%) *Including Covid-19*	111 (75.5) *58 (39.5)*	63 (75.0) *36 (42.9)*	48 (76.2) *22 (34.6)*	1.000 *0.422*
**Ventilatory parameters at ARDS onset (day 1 of invasive ventilation)**				
FiO_2_, %	100 [80–100]	100 [100–100]	100 [68–100]	0.038
Tidal volume, mL/kg PBW	6.1 [5.8–6.5]	6.1 [5.8–6.7]	6.0 [5.8–6.3]	0.386
Respiratory rate, breaths/min	26 [24–28]	26 [24–30]	26 [24–28]	0.267
PEEP, cmH_2_O	10 [6–12]	10 [6–12]	8 [6–10]	0.296
Driving pressure, cmH_2_O	13 [11–16]	13 [11–17]	14 [11–16]	0.799
Crs, mL/cmH_2_O	31 [26–38]	32 [25–39]	31 [27–37]	0.766
PaO_2_ on FiO_2_ ratio	111 [86–163]	107 [87–159]	120 [83–172]	0.421
Ventilatory ratio	1.78 [1.55–2.07]	1.79 [1.63–2.16]	1.71 [1.43–1.97]	0.002
**Respiratory mechanics at NBMA weaning**				
Plateau pressure, cmH_2_O	24 [21–26]	24 [22–26]	23 [20–25]	0.085
Driving pressure, cmH_2_O	12 [10–14]	12 [10–14]	12 [10–14]	0.564
Crs, mL/cmH_2_O	34 [28–41]	34 [28–41]	34 [28–42]	0.415
Rrs, cmH_2_O/L/s	13 [10–17]	13 [10–17]	13 [11–15]	0.689
Mechanical power, J/min	26.4 [21.2–35.1]	29.9 [22.3–36.4]	24.7 [20.9–29.3]	0.073
**Ventilatory parameters in ACV and blood gases before PSV initiation**				
FiO_2_, %	50 [40–50]	50 [40–60]	45 [40–50]	0.136
Tidal volume, mL/kg PBW	6.1 [5.7–6.6]	6.2 [5.7–6.8]	6.0 [5.7–6.4]	0.440
RR, breaths/min	29 [25–33]	30 [26–33]	28 [25–32]	0.157
Minute ventilation, L/min	11.8 [10.2–13.4]	12.1 [10.6–14.1]	11.2 [9.9–12.8]	0.128
PEEP, cmH_2_O	10 [8–12]	10 [8–12]	10 [8–12]	0.588
pH	7.41 [7.36–7.45]	7.39 [7.33–7.44]	7.43 [7.38–7.47]	0.002
PaCO_2_, mmHg	44 [39–49]	44 [40–50]	42 [39–47]	0.055
PaO_2_ on FiO_2_ ratio	178 [148–230]	170 [132–220]	189 [154–248]	0.099
HCO_3_, mmol/L	27.6 [24.1–31.0]	27.0 [24.0–31.1]	28.0 [25.0–31.0]	0.918
Mean ventilatory parameters during PSV*				
FiO_2_, %	50 [40–60]	50 [45–60]	45 [40–50]	0.001
PSL, mmHg	10 [10–12]	12 [10–12]	10 [10–12]	0.705
Tidal volume, mL	507 [420–605]	509 [434–660]	497 [394–567]	0.015
Tidal volume, mL/kg PBW	7.5 [6.4–8.8]	8.0 [6.7–9.6]	7.3 [6.0–8.3]	0.002
RR, breaths/min	24 [19–29]	23 [19–29]	24 [20–28]	0.711
Minute ventilation, L/min	11.5 [9.7–14.2]	11.5 [10.0–14.7]	11.5 [9.5–13.3]	0.387
PEEP, cmH_2_O	8 [6–10]	8 [6–10]	8 [6–8]	0.016
Time spent under PSV, hours	27 [6–51]	9 [6–24]	51 [42–60]	< 0.001
**Sedation management during PSV**				
Continuous sedation at PSV initiation, *n* (%)	103 (70.1)	65 (77.4)	38 (60.3)	0.040
Continuous sedation 24 h after PSV initiation, *n* (%)	74 (54.8)	59 (72.8)	15 (27.8)	< 0.001
Sedation trajectory:				< 0.001
Persistent sedation Sedation discontinued Maintained without sedation Sedation reinitiated	66 (48.9) 29 (21.5) 32 (23.7) 8 (5.9)	53 (65.4) 9 (11.1) 13 (16.0) 6 (7.4)	13 (24.1) 20 (37.0) 19 (35.2) 2 (3.7)	

* Parameters during PSV were averaged from data collected at three-hour intervals throughout the time spent in PSV

*Abbreviations*: Crs: compliance of the respiratory system; Rrs: resistance of the respiratory system; PSV: pressure support ventilation; ARDS: acute respiratory distress syndrome; NMBA: neuromuscular blocking agent; VCV: volume-controlled ventilation; PEEP: positive end-expiratory pressure; PSL: pressure support level; PBW: predicted body weight

**Fig. 3:**
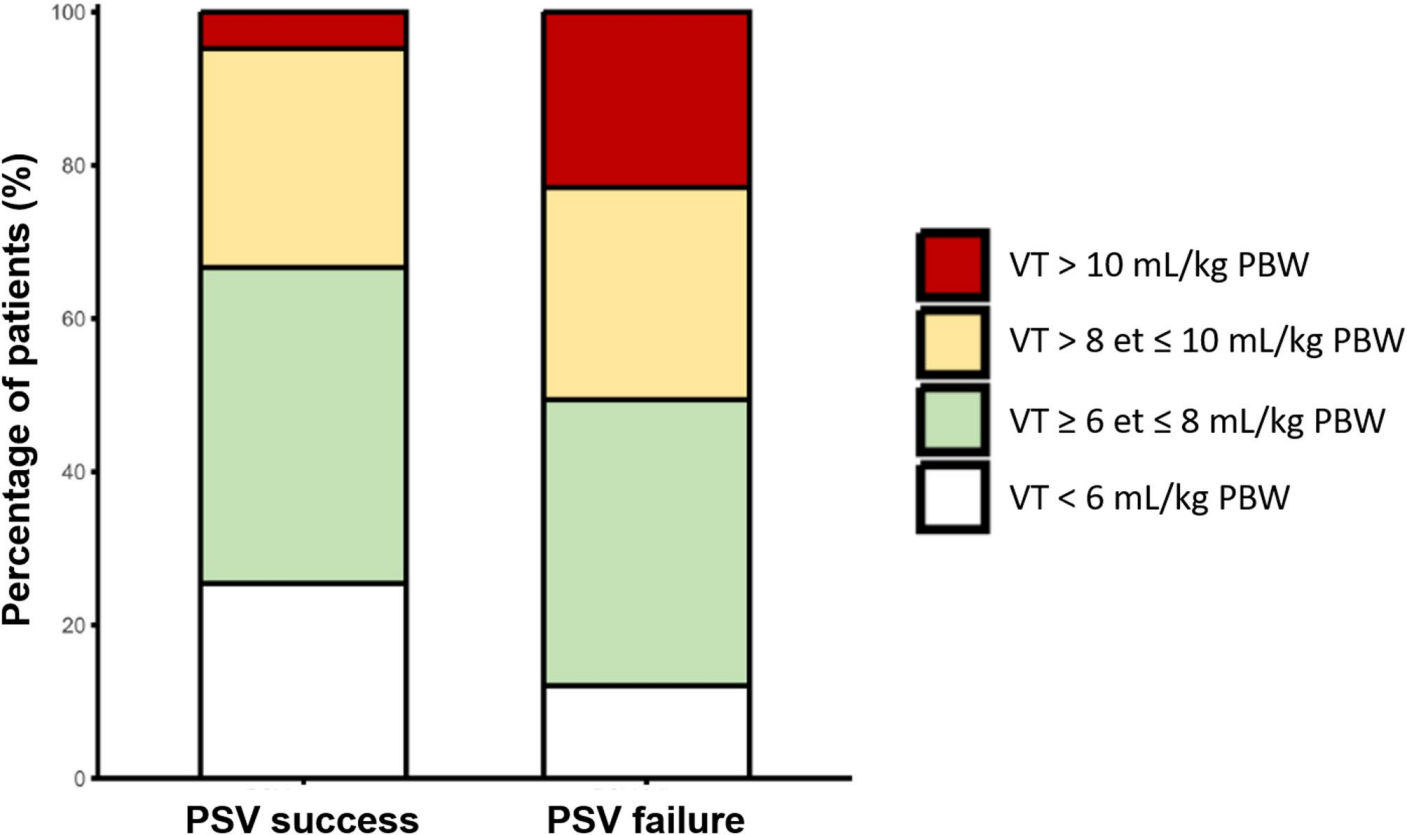
Distribution of tidal volume during PSV. Relative distribution of mean tidal volume during PSV according to predefined ranges in patients who stayed in PSV (PSV success) and in patients who needed to be switched back to ACV (PSV failure) during the transition phase *Abbreviations*: PSV: pressure support ventilation; VT: tidal volume; PBW: predicted body weight; ACV; assisted controlled ventilation

Ventilatory parameters at the initiation of PSV and during PSV are shown in Table 4. During PSV, 57 patients (39%) had a tidal volume between 6 and 8 mL/kg of PBW, 41 patients (28%) had a tidal volume between 8 and 10 mL/kg of PBW, and 22 patients (15%) had a tidal volume greater than 10 mL/kg of PBW, of whom 19 (86%) failed PSV (Fig. 3). In patients who failed PSV, the comparison of ventilatory parameters between PSV initiation and just before PVS failure is shown Table E5.

Variables associated with PSV failure by univariate analysis included pH before PSV initiation, FiO_2_, PEEP and tidal volume during PSV, the time spent under PSV and continuous sedation (Table 5). Multivariate analysis identified tidal volume as factor independently associated with PSV failure (Table 5).

**Table 5:** Multivariate analysis of risk factors for pressure support ventilation failure

	Univariate			Multivariate		
	OR	95% IC	*p*-value	OR	95% IC	*p*-value
SOFA at PSV initiation	1.01	0.92–1.11	0.852	-		
Sedation at PSV initiation	2.25	1.10–4.67	0.027	1.62	0.73–3.64	0.236
Mean ventilatory parameters during PSV*						
FiO_2_, %	1.05	1.02–1.09	0.001	1.03	1.00–1.07	0.087
Tidal volume, mL/kg PBW	1.30	1.10–1.57	0.003	1.28	1.06–1.56	0.012
Respiratory rate, breaths/min	0.99	0.95–1.04	0.709	-		
PSL, cmH_2_O	1.03	0.90–1.18	0.703	-		
PEEP, cmH_2_O	1.22	1.04–1.43	0.017	1.19	1.00–1.44	0.056
**Blood gases in ACV before PSV initiation**						
pH	0.47	0.28–0.76	0.003	0.60	0.34–1.03	0.068
PaO_2_, mmHg	1.00	0.99–1.01	0.740	-		
PaCO_2_, mmHg	1.04	1.00–1.08	0.062	-		
PaO_2_/FiO_2_, mmHg	1.00	0.99–1.00	0.103	-		
Bicarbonate, mmol/L	1.00	0.94–1.07	0.918	-		

*The mean values for ventilatory variables on PSV were calculated as the average of the data collected at three-hour intervals

*Abbreviations*: SOFA: sepsis-related Organ Failure Assessment; PSV: pressure support ventilation; PBW: predicted body weight; PSL: pressure support level; PEEP: positive end-expiratory pressure; ACV: assisted controlled ventilation

Using ROC curve analysis, the optimal threshold for predicting the failure of PSV was a tidal volume of 9.3 ml/kg of PBW, with low sensitivity of (30%) but high specificity of (91%) (Supplemental Material, Figure E2).

### Transition phase variables associated with the outcome

28-day mortality was 19% (*n* = 38) in our cohort, and ventilator-free days at day 28 were 7 days [0.0–19.0] in median. Mortality was significantly higher in the NMBA weaning failure group (23 deaths (31%) vs. 15 (12%), *p* = 0.003) but not in the PSV failure group (21 deaths (25%) vs. 7 (11%), *p* = 0.062). Ventilator-free days at day 28 were significantly lower in both the NMBA weaning failure group (0.0 [0.0–9.0] days vs. 15.0 [0.0–21.8], *p* < 0.001) and the PSV failure group (0.0 [0.0–16.5] days vs. 18.0 [8.5–23.0], *p* < 0.001).

NMBA weaning failure was independently associated with mortality (OR 3.70 [1.56–9.16], *p* = 0.004) and ventilator-free days at day 28 (HR 1.93 [1.22–3.04], *p* = 0.005) (Tables E1 and E2). In contrast, the absence of a switch to PSV during the transition phase was not independently associated with outcomes at day 28 (Tables E1 and E2). However, PSV failure was independently associated with fewer ventilator-free days at day 28 (HR 1.98 (1.27–3.09), *p* = 0.003) (Table E4).

Tidal volume during PSV was independently associated with mortality at day 28 (OR 1.37 [1.07–1.78], *p* = 0.016) (Table E3). Using ROC curve analysis, the accuracy of tidal volume in predicting mortality at day 28 was acceptable, with area under the curve (AUC) value of 0.731. According to the Youden index, the optimal threshold for predicting mortality at day 28 was identified as a tidal volume of 8.9 mL/kg of PBW (sensitivity 61% and specificity 84%) (Supplemental Material, Figure E3).

Forty-one patients failed both NMBA weaning and PSV: 33 patients initially failed PSV and underwent NMBA resumption after switching back to volume-controlled ventilation, and 9 patients initially underwent NMBA resumption and then failed PSV after a second NMBA withdrawal (see Fig. 1). These 41 patients were not different from the rest of the cohort in terms of demographic characteristics or ARDS severity at intubation or at the onset of the transition phase. The only significant differences were a lower driving pressure at intubation (12 [10–15] cmH_2_O vs. 14 [11–17], *p* = 0.025) and a lower pH at the onset of the transition phase (7.38 [7.31–7.41] vs. 7.40 [7.34–7.45], *p* = 0.017) in the subgroup of patients who failed both NMBA weaning and PSV. The 28-day mortality was higher in this subgroup compared to the rest of the cohort (34% vs. 16%, *p* = 0.014). Similarly, 28-day ventilator-free days were lower (0.0 [0.0–9.2] days vs. 12.0 [0.0–20.0], *p* < 0.001).

## Discussion

This study assesses the critical challenges associated with the transition phase in moderate-to-severe ARDS. All patients required continuous neuromuscular blockade, and 51% were placed in the prone position before the onset of the transition phase. The 96 patients who remained in the supine position were those who improved sufficiently with protective ventilation provided by NMBA during a stabilization period of 12 to 24 h, no longer meeting the criteria for prone position thereafter [22]. Among the 196 patients included, 62% successfully discontinued NMBA after the first attempt, and 75% transitioned to assisted ventilation. However, the high failure rates for both NMBA weaning (38%) and PSV trials (57%) underscore the complexity of managing this intermediate phase.

### NBWA weaning

The use of NMBAs in ARDS remains a subject of ongoing debate [4], with continuous infusion of cisatracurium for 48 h being the only regimen shown to improve survival [7]. Despite this, growing awareness of the risks associated with prolonged NMBA use, including ICU-acquired weakness [23, 24] and their uncertain benefit [6], has led to tend toward a more individualized approach [3]. However, the lack of standardized criteria for NMBA discontinuation poses a significant challenge, as reflected in the variability of practices reported in prior studies [8]. Our findings are among the first to identify factors for NMBA weaning failure in moderate-to-severe ARDS.

Interestingly, respiratory mechanics, assessed through driving pressure, were not associated with NMBA weaning failure when analyzed continuously. However, categorization into low (< 12 cmH_2_O), intermediate (12–13 cmH_2_O), and high (≥ 14 cmH_2_O) driving pressure ranges revealed a significant association between extreme values and NMBA weaning failure requiring reintroduction. High driving pressure, which likely reflects overdistension and poor compliance, has been shown to be associated with increased mortality in ARDS [1, 25–27]. At the time of NMBA weaning attempts, it may indicate persistent, severe lung injury. Conversely, it is unclear whether further reducing driving pressure below a certain threshold (< 10–12 cmH_2_O) is beneficial [25, 27]. Patients with low driving pressure typically exhibit higher respiratory system compliance. In such cases, a tidal volume set at 6 mL/kg of PBW might be disproportionately low related to the available aerated lung volume [28–30], potentially causing air hunger during NMBA and sedation weaning. This could lead to poor patient-ventilator interactions, with a high respiratory drive and asynchronies contributing to discomfort, poor tolerance, and potential PSILI [31].

Our study also identified pH and PaO_2_/FiO_2_ ratio as modifiable predictors of NMBA weaning failure. Although the overall predictive performance of pH and PaO_2_/FiO_2_ for NMBA weaning failure is limited, thresholds with high specificity may help identify patients at high risk of failure. For example, pronounced hypoxemia (PaO_2_/FiO_2_ < 150 mmHg) or acidemia (pH < 7.30) may serve as warning signals against premature NMBA withdrawal. The association between COVID-19 and NMBA weaning failure may reflect distinct pathophysiological features of COVID-19-related ARDS [32], although subgroup analyses did not reveal significant differences in predictive factors (Table E6 and Table E7).

In our study, NMBA weaning failure was significantly associated with prolonged mechanical ventilation and increased mortality at day 28. The observational nature of the study precludes the establishment of a causal relationship and this association warrants further investigation in future studies.

In our study, NMBA weaning failure was defined as NMBA resumption within 48 h following withdrawal. This threshold was chosen arbitrarily and may be subject to debate. When considering the entire observation period (i.e., 72 h), an additional 6 patients required NMBA resumption (for a total of 82 patients). A sensitivity analysis was performed (not reported), including these 6 patients, and did not alter the study results, neither for the predictive factors of failure nor for the association between failure and outcomes. We deliberately maintained this early 48-hour threshold to focus on NMBA weaning failures related to premature NMBA withdrawal.

### Pressure support ventilation

Transitioning to PSV during the transition phase was successful in 43% of patients, with a failure rate as high as 57%. PSV failure was associated with fewer ventilator-free days at day 28, highlighting the transition phase as a critical area for future research to improve patient outcomes. In a cohort of 100 unselected patients, including approximately 30% with ARDS, the failure rate of PSV during the first 48 h was 22% [33]. In our series, despite moderate inspiratory support and normal pH at PSV initiation, 43% of patients exhibited high tidal volumes (> 8 mL/kg PBW). Higher tidal volumes were associated with increased mortality. Higher tidal volumes were likely a consequence of increased respiratory effort. However, respiratory drive or effort was not measured in our cohort. In a series of 28 COVID-19 patients, elevated P0.1 values were predictive of PSV failure during the transition phase [34]. These findings underscore the importance of closely monitoring tidal volume and respiratory drive or effort during this critical phase in the management of ARDS.

### Limitations

Our study has several limitations, primarily due to its retrospective nature. Some data, such as detailed respiratory mechanics at the time of PSV initiation, asynchronies and measures of patient effort (e.g., esophageal pressure swings) or respiratory drive (e.g., P0.1) were not available. However, the granularity of the collected data, such as tidal volume recorded every three-hours, is a unique strength of this study. The reintroduction of NMBA and the return from PSV to ACV, which defined NMBA weaning failure and PSV failure respectively, were not standardized across centers. However, no significant center effect was identified in our analysis. Similarly, the reasons for NMBA resumption were not documented in the medical records for one-third of the patients. It is possible that, in addition to the mentioned causes (hypoxemia, asynchronies, respiratory distress, prone positioning), impaired respiratory mechanics (elevated driving pressure or plateau pressure) may have also prompted attending physicians to resume NMBA in some of these patients.

Since the transition phase is poorly described in the literature, we adopted an arbitrary definition as the 72-hour period following the first NMBA weaning attempt. This choice may limit the generalizability of our findings to ARDS cases without continuous NMBA infusion. The alternative entry point for defining the transition phase in ARDS could be the switch from controlled to assisted ventilation, which has been evaluated in our study.

## Conclusion

This study identifies the transition phase as a high-risk period in ARDS, with significant failure rates for NMBA weaning and PSV trials that may influence patient outcomes. In our cohort, risk factors associated with NMBA weaning failure included high or low driving pressure, lower pH, and reduced PaO_2_/FiO_2_ ratio. Similarly, risk factor associated with PSV failure was high tidal volumes. The transition phase therefore represents a critical area for future research to optimize patient management and improve outcomes, and further prospective studies are warranted to develop strategies that optimize management during this vulnerable period.

## Electronic supplementary material

Below is the link to the electronic supplementary material.

Supplementary Material 1

## Data Availability

The datasets used and/or analyzed during the current study are available from the corresponding author on reasonable request.

## Declarations

### Descriptor number

4.8 Mechanical Ventilation: Physiology & Pathophysiology.

## Abbreviations

ACV: Assist-control ventilation
ARDS: Acute respiratory distress syndrome
ICU: Intensive care unit
LOS: Length of stay
NIV: Non-invasive ventilation
NMBA: Neuromuscular blocking agent
PBW: Predicted body weight
PEEP: Positive end-expiratory pressure
P-SILI: Patient self-inflected lung injury
PSL: Pressure support level
PSV: Pressure support ventilation
ROC: Receiver-operating characteristic
SAPS II: Simplified acute physiology score II
SOFA: Sepsis-related Organ Failure Assessment
VFD: Ventilator-free days
