# Supplementary material for: Factors influencing the transition phase in acute respiratory distress syndrome: an observational cohort study
Source: Ann Intensive Care. 2025 May 26;15:71. doi: 10.1186/s13613-025-01484-6 (PMC12104120; doi:10.1186/s13613-025-01484-6)

**ELECTRONIC SUPPLEMENTARY MATERIAL**

# Factors influencing the transition phase in acute respiratory distress syndrome: an observational cohort study.

# Anne-Fleur HAUDEBOURG^1,2^ M.D., Louise CHANTELOT^1^ M.D., Safaa NEMLAGHI^1^ M.D., Luc HAUDEBOURG^4^ M.D., Pascale LABEDADE^1,2^ M.D., Mohamed Ahmed BOUJELBEN^1,2^ M.D., Guillaume VOIRIOT^5^, M.D., Ph.D., Armand MEKONTSO DESSAP^1,2,3^ M.D., Ph.D., Muriel FARTOUKH^5^, M.D., Ph.D., Guillaume CARTEAUX^1,2,3^ M.D., Ph.D.

**Table E1: multivariate analysis of risk factors for D28 mortality at first withdrawal of neuromuscular blockers**

|  | **Univariate** | | | **Multivariate** | | |
| --- | --- | --- | --- | --- | --- | --- |
|  | **OR** | **95% IC** | **p-value** | **OR** | **95% IC** | **p-value** |
| NMBA weaning failure | 3.19 | 1.55 - 6.74 | 0.002 | 3.70 | 1.56 - 9.16 | 0.004 |
| Switch to PSV during transition phase | 0.93 | 0.42 - 2.16 | 0.851 | - |  |  |
| **Demographic characteristics** | | | | | | |
| Age, years | 1.08 | 1.04 - 1.12 | < 0.001 | - |  |  |
| SAPS II at ICU admission | 1.05 | 1.02 - 1.08 | < 0.001 | 1.02 | 0.99 - 1.06 | 0.179 |
| Comorbidities:  Chronic heart failure  Chronic kidney failure  COPD  Immunosuppression,  Malignancy | 1.93  2.01  0.67  1.96  2.01 | [0.50 - 6.33]  [0.80 - 4.74]  [0.10 - 2.61]  [0.84 - 4.36]  [0.80 - 4.74] | 0.295  0.119  0.612  0.105  0.119 | -  -  -  -  - |  |  |
| **ARDS cause** | | | | | | |
| Pulmonary ARDS | 4.40 | 1.48 - 18.92 | 0.018 | 4.62 | 1.32 - 22.68 | 0.031 |
| Covid-19 | 0.59 | 0.30 - 1.13 | 0.116 | - |  |  |
| **Characteristics at NMBA weaning** | | | | | | |
| SOFA | 1.28 | 1.15 - 1.43 | < 0.001 | 1.19 | 1.03 - 1.39 | 0.02 |
| Vasopressors | 2.75 | 1.31 - 6.15 | 0.010 | - |  |  |
| RRT | 1.54 | 0.52 - 4.05 | 0.398 | - |  |  |
| Tidal volume, mL/kg PBW | 1.19 | 0.68 - 2.09 | 0.541 | - |  |  |
| RR, breaths/min | 1.06 | 0.98 - 1.14 | 0.147 | - |  |  |
| Minute ventilation, L/min | 1.32 | 1.13 - 1.56 | 0.001 | 1.30 | 1.09 - 1.58 | 0.005 |
| PEEP, cmH_2_O | 0.89 | 0.78 - 1.01 | 0.072 | 0.81 | 0.68 - 0.94 | 0.007 |
| Driving pressure | 1.01 | 0.93 - 1.11 | 0.748 | - |  |  |
| pH | 0.59 | 0.37 - 0.92 | 0.021 | 1.22 | 0.69 - 2.21 | 0.494 |
| PaO_2_/FiO_2_, mmHg | 0.96 | 0.90 - 1.01 | 0.114 | - |  |  |
| Ventilatory ratio | 1.04 | 0.98 - 1.11 | 0.143 | - |  |  |

*Abbreviations: ICU: intensive care unit; NMBA: neuromuscular blocking agent; SAPS II: simplified acute physiology score II; SOFA: sepsis-related Organ Failure Assessment; ARDS: acute respiratory distress syndrome; RRT: renal replacement therapy; PEEP: positive end-expiratory pressure; PBW: predicted body weight*

**Table E2: multivariate analysis of risk factors for VFD28 at first withdrawal of neuromuscular blockers**

|  | **Univariate** | | | **Multivariate** | | |
| --- | --- | --- | --- | --- | --- | --- |
|  | **HR** | **95% IC** | **p-value** | **HR** | **95% IC** | **p-value** |
| NMBA weaning failure | 2.14 | 1.49 - 3.07 | < 0.001 | 1.93 | 1.22 - 3.04 | 0.005 |
| Switch to PSV during transition phase | 0.65 | 0.45 - 0.95 | 0.027 | 0.93 | 0.58 - 1.50 | 0.770 |
| **Demographic characteristics** | | | | | | |
| Age, years | 1.03 | 1.02 - 1.05 | < 0.001 | - |  |  |
| SAPS II at ICU admission | 1.02 | 1.01 - 1.04 | 0.001 | 1.02 | 1.00 - 1.03 | 0.096 |
| Covid-19 | 1.67 | 1.16 - 2.38 | 0.005 | 1.67 | 1.10 - 2.54 | 0.016 |
| **Characteristics at NMBA weaning** | | | | | | |
| SOFA | 1.13 | 1.07 - 1.20 | < 0.001 | 1.08 | 1.01 - 1.16 | 0.031 |
| Vasopressors | 1.28 | 0.91 - 1.82 | 0.160 | - |  |  |
| RRT | 2.13 | 1.10 - 4.09 | 0.024 | - |  |  |
| Tidal volume, mL/kg PBW | 1.26 | 0.97 - 1.65 | 0.086 | - |  |  |
| RR, breaths/min | 1.05 | 1.01 - 1.09 | 0.016 | 1.00 | 0.96 - 1.05 | 0.980 |
| Minute ventilation, L/min | 1.15 | 1.06 - 1.24 | 0.001 | 1.07 | 0.94 - 1.22 | 0.310 |
| PEEP, cmH_2_O | 0.95 | 0.89 - 1.00 | 0.051 | - |  |  |
| Driving pressure | 1.07 | 1.02 - 1.13 | 0.010 | 1.09 | 1.03 - 1.16 | 0.002 |
| pH | 0.66 | 0.52 - 0.83 | < 0.001 | 0.79 | 0.61 - 1.02 | 0.071 |
| PaO_2_/FiO_2_, mmHg | 0.97 | 0.95 - 1.00 | 0.042 | - |  |  |
| Ventilatory ratio | 1.06 | 1.03 - 1.10 | 0.001 | - |  |  |

*Abbreviations: VFD28: ventilator-free days at day 28; ICU: intensive care unit; NMBA: neuromuscular blocking agent; SAPS II: simplified acute physiology score II; SOFA: sepsis-related Organ Failure Assessment; ARDS: acute respiratory distress syndrome; RRT: renal replacement therapy; PEEP: positive end-expiratory pressure; PBW: predicted body weight*

**Table E3: multivariate analysis of risk factors for D28 mortality at pressure support ventilation initiation**

|  | **Univariate** | | | **Multivariate** | | |
| --- | --- | --- | --- | --- | --- | --- |
|  | **OR** | **95% IC** | **p-value** | **OR** | **95% IC** | **p-value** |
| PSV failure | 2.62 | 1.08 - 7.08 | 0.042 | 1.10 | 0.27 - 4.60 | 0.894 |
| **Demographic characteristics** | | | | | | |
| Age, years | 1.08 | 1.04 - 1.12 | < 0.001 | - |  |  |
| SAPS II at ICU admission | 1.05 | 1.02 - 1.08 | < 0.001 | 1.02 | 0.98 - 1.06 | 0.302 |
| **ARDS cause** | | | | | | |
| Pulmonary ARDS | 4.40 | 1.48 - 18.92 | 0.018 | 2.09 | 0.58 - 10.12 | 0.298 |
| Covid-19 | 0.59 | 0.30 - 1.13 | 0.116 | - |  |  |
| **Characteristics in ACV before PSV initiation** | | | | | | |
| SOFA | 1.27 | 1.13 - 1.45 | < 0.001 | 1.17 | 1.00 - 1.40 | 0.061 |
| Vasopressors | 3.40 | 1.43 - 8.78 | 0.008 | - |  |  |
| RRT | 1.54 | 0.52 - 4.05 | 0.398 | - |  |  |
| Minute ventilation, L/min | 1.34 | 1.14 - 1.63 | 0.001 | - |  |  |
| FiO_2_, % | 1.04 | 1.01 - 1.06 | 0.004 | - |  |  |
| pH | 0.39 | 0.21 - 0.68 | 0.001 | 0.52 | 0.26 - 1.01 | 0.059 |
| Ventilatory ratio | 1.08 | 1.01 - 1.16 | 0.022 | - |  |  |
| **Mean ventilatory parameters during PSV*** | | | | | | |
| Time spent in PSV, hours | 0.98 | 0.96 - 1.00 | 0.059 | 0.99 | 0.96 - 1.02 | 0.701 |
| FiO_2_, % | 1.04 | 1.01 - 1.07 | 0.013 | - |  |  |
| Tidal volume, mL/kg PBW | 1.53 | 1.26 - 1.91 | < 0.001 | 1.37 | 1.07 - 1.78 | 0.016 |
| Respiratory rate, breaths/min | 0.93 | 0.87 - 0.99 | 0.027 | 0.98 | 0.90 - 1.07 | 0.667 |
| PSL, cmH_2_O | 0.89 | 0.75 - 1.06 | 0.203 | - |  |  |
| PEEP, cmH_2_O | 1.00 | 0.83 - 1.21 | 0.967 | - |  |  |

**Parameters on PSV were averaged based on data collected every 3 hours during time spent in PSV.*

*Abbreviations: ICU: intensive care unit; ACV: assisted controlled ventilation; PSV: pressure support ventilation; SAPS II: simplified acute physiology score II; SOFA: sepsis-related Organ Failure Assessment; ARDS: acute respiratory distress syndrome; PEEP: positive end-expiratory pressure; PBW: predicted body weight*

**Table E4: multivariate analysis of risk factors for VFD28 at pressure support ventilation initiation**

|  | **Univariate** | | | **Multivariate** | | |
| --- | --- | --- | --- | --- | --- | --- |
|  | **HR** | **95% IC** | **p-value** | **HR** | **95% IC** | **p-value** |
| PSV failure | 2.30 | 1.55 - 3.42 | < 0.001 | 1.98 | 1.27 - 3.09 | 0.003 |
| **Demographic characteristics** | | | | | | |
| Age, years | 1.03 | 1.02 - 1.05 | < 0.001 | - |  |  |
| SAPS II at ICU admission | 1.02 | 1.01 - 1.04 | 0.001 | 1.02 | 1.00 - 1.04 | 0.052 |
| Covid-19 | 1.67 | 1.16 - 2.38 | 0.005 | 1.90 | 1.20 - 3.00 | 0.006 |
| **Characteristics in ACV before PSV initiation** | | | | | | |
| SOFA | 1.12 | 1.05 - 1.18 | < 0.001 | 1.06 | 0.98 - 1.15 | 0.120 |
| Vasopressors | 1.67 | 1.11 - 2.51 | 0.014 | - |  |  |
| RRT | 2.13 | 1.10 - 4.09 | 0.024 | - |  |  |
| Minute ventilation, L/min | 1.19 | 1.09 - 1.29 | < 0.001 | 1.08 | 0.97 - 1.21 | 0.140 |
| PaO_2_/FiO_2_, mmHg | 0.95 | 0.93 - 0.97 | < 0.001 | - |  |  |
| pH | 0.57 | 0.44 - 0.74 | < 0.001 | 0.79 | 0.58 - 1.08 | 0.140 |
| Ventilatory ratio | 1.09 | 1.05 - 1.14 | < 0.001 | - |  |  |
| **Mean ventilatory parameters during PSV*** | | | | | | |
| Fi_2_O, % | 1.04 | 1.02 - 1.06 | < 0.001 | - |  |  |
| Tidal volume, mL/kg PBW | 1.20 | 1.09 - 1.32 | < 0.001 | 1.06 | 0.94 - 1.19 | 0.320 |
| RR, breaths/min | 0.98 | 0.96 - 1.01 | 0.210 | - |  |  |
| PSL, cmH_2_O | 1.01 | 0.94 - 1.09 | 0.830 | - |  |  |
| PEEP, cmH_2_O | 0.97 | 0.88 - 1.07 | 0.530 | - |  |  |

**Parameters on PSV were averaged based on data collected every 3 hours during time spent in PSV.*

*Abbreviations: VFD28: ventilator-free days at day 28; ICU: intensive care unit; PSV: pressure support ventilation; SAPS II: simplified acute physiology score II; SOFA: sepsis-related Organ Failure Assessment; ARDS: acute respiratory distress syndrome; RRT: renal replacement therapy; PSL: pressure support level; PEEP: positive end-expiratory pressure; PBW: predicted body weight*

**Table E5: In patients with PSV failure, comparison of ventilatory parameters and respiratory mechanics between PSV initiation and just before failure**

|  | **PSV initiation** | **Before PSV failure** | **p-value** |
| --- | --- | --- | --- |
| FiO_2_, % | 50 [45 - 60] | 55 [45 - 67] | 0.038 |
| Tidal volume, mL | 509 [435 - 661] | 484 [420 - 589] | 0.004 |
| Tidal volume, mL/kg PBW | 8.0 [6.7 - 9.5] | 7.3 [6.1 - 9.2] | 0.005 |
| Respiratory rate, breaths/min | 23 [19 - 29] | 24 [20 - 31] | 0.026 |
| Minute ventilation, L/min | 11.5 [10.0 - 14.7] | 12.1 [9.7 - 14.8] | 0.559 |
| PSL, cmH_2_O | 12 [10 - 12] | 12 [10 - 13] | 0.526 |
| PEEP, cmH_2_O | 8 [6 - 10] | 8 [6 - 10] | 0.163 |
| Systolic blood pressure, mmHg | 129 [115 - 147] | 133 [115 - 150] | 0.510 |
| Diastolic blood pressure, mmHg | 61 [55 - 73] | 60 [55 - 72] | 0.703 |
| Mean blood pressure, mmHg | 82 [74 - 97] | 82 [73 - 95] | 0.963 |
| Heart rate, bpm | 89 [76 - 102] | 89 [79 - 107] | 0.409 |

*Abbreviations: PSL: pressure support level; PEEP: positive end-expiratory pressure; PBW: predicted body weight*

**Table E6: ventilatory parameters and respiratory mechanics at first neuromuscular blockade weaning attempt in non-Covid ARDS**

|  | **All patients**  **n = 109** | **NMBA weaning failure**  **n = 29** | **NMBA weaning success**  **n = 80** | **p-value** |
| --- | --- | --- | --- | --- |
| **Ventilatory parameters** | | | | |
| FiO_2_, % | 50 [50 - 70] | 60 [50 - 70] | 50 [50 - 60] | 0.311 |
| Tidal volume, mL | 405 [364 - 445] | 413 [376 - 464] | 402 [364 - 440] | 0.150 |
| Tidal volume, mL/kg PBW | 6.1 [5.7 - 6.3] | 6.2 [5.9 - 6.6] | 6.0 [5.7 - 6.3] | 0.054 |
| Respiratory rate, breaths/min | 30 [25 - 32] | 30 [26 - 34] | 29 [25 - 32] | 0.257 |
| Minute ventilation, L/min | 11.5  [10.1 - 13.3] | 12.0  [10.3 - 14.2] | 11.4  [10.0 - 12.9] | 0.118 |
| Peak pressure, cmH_2_O | 37 [32 - 41] | 37 [33 - 40] | 36 [32 - 41] | 0.646 |
| Plateau pressure, cmH_2_O | 24 [20 - 27] | 24 [21 - 28] | 24 [20 - 27] | 0.441 |
| PEEP, cmH_2_O | 10 [8 - 14] | 12 [10 - 15] | 10 [8 - 14] | 0.108 |
| Driving pressure, cmH_2_O | 12 [10 - 14] | 11 [10 - 14] | 12 [10 - 14] | 0.703 |
| Crs, mL/cmH_2_O | 34 [27 - 42] | 35 [27 - 43] | 34 [27 - 39] | 0.669 |
| Mechanical power, J/min | 26.1  [21.1 - 33.4] | 28.0  [22.8 - 33.8] | 25.9  [20.1 - 32.5] | 0.140 |
| **Blood gases** | | | | |
| pH | 7.40  [7.31 - 7.45] | 7.36  [7.29 - 7.41] | 7.40  [7.34 - 7.46] | 0.006 |
| PaCO_2_, mmHg | 42 [37 - 49] | 43 [38 - 51] | 41 [36 - 48] | 0.085 |
| PaO_2_, mmHg | 103 [83 - 152] | 121 [87 - 141] | 99 [81 - 160] | 0.486 |
| PaO_2_ on FiO_2_ ratio | 196 [158 - 250] | 196 [174 - 218] | 196 [158 - 270] | 0.131 |
| Bicarbonates, mmol/L | 26.5  [23.5 - 30.0] | 27.0  [23.5 - 30.0] | 26.4  [23.5 - 30.1] | 0.765 |
| Lactates, mmol/L | 1.4 [1.1 - 2.0] | 1.4 [1.1 - 1.7] | 1.5 [1.0 - 2.0] | 0.464 |

*Abbreviations: NMBA: neuromuscular blocking agent; PEEP: positive end-expiratory pressure; Crs: compliance of the respiratory system*

**Table E7: ventilatory parameters and respiratory mechanics at first neuromuscular blockade weaning attempt in Covid ARDS**

|  | **All patients**  **n = 87** | **NMBA weaning failure**  **n = 45** | **NMBA weaning success**  **n = 42** | **p-value** |
| --- | --- | --- | --- | --- |
| **Ventilatory parameters** | | | | |
| FiO_2_, % | 50 [40 - 50] | 50 [40 - 60] | 40 [40 - 50] | 0.003 |
| Tidal volume, mL | 422 [384 - 452] | 427 [399 - 450] | 417 [372 - 457] | 0.860 |
| Tidal volume, mL/kg PBW | 6.2 [5.9 - 6.6] | 6.2 [5.9 - 6.5] | 6.2 [5.9 - 6.7] | 0.898 |
| Respiratory rate, breaths/min | 28 [24 - 30] | 30 [25 - 32] | 28 [23 - 30] | 0.090 |
| Minute ventilation, L/min | 11.1  [9.9 - 12.9] | 11.3  [10.5 - 13.7] | 10.9  [9.6 - 12.4] | 0.196 |
| Peak pressure, cmH_2_O | 38 [35 - 41] | 39 [35 - 43] | 38 [34 - 40] | 0.456 |
| Plateau pressure, cmH_2_O | 24 [21 - 26] | 24 [22 - 26] | 23 [21 - 25] | 0.446 |
| PEEP, cmH_2_O | 10 [10 - 12] | 10 [10 - 12] | 10 [10 - 12] | 0.361 |
| Driving pressure, cmH_2_O | 12 [11 - 15] | 12 [10 - 15] | 12 [11 - 15] | 0.698 |
| Crs, mL/cmH_2_O | 33 [27 - 41] | 33 [26 - 43] | 33 [27 - 40] | 0.658 |
| Mechanical power, J/min | 26.9  [22.5 - 35.2] | 27.0  [23.1 - 36.4] | 25.9  [19.2 - 33.1] | 0.198 |
| **Blood gases** | | | | |
| pH | 7.40  [7.35 - 7.42] | 7.38  [7.34 - 7.42] | 7.40  [7.37 - 7.43] | 0.027 |
| PaCO_2_, mmHg | 44 [41 - 48] | 44 [42 - 49] | 44 [41 - 48] | 0.314 |
| PaO_2_, mmHg | 93 [75 - 114] | 90 [75 - 109] | 94 [77 - 115] | 0.820 |
| PaO_2_ on FiO_2_ ratio | 192 [164 - 235] | 186 [154 - 210] | 199 [185 - 253] | 0.014 |
| Bicarbonates, mmol/L | 27.0  [23.5 - 30.0] | 25.9  [22.0 - 30.0] | 27.0  [25.0 - 29.9] | 0.601 |
| Lactates, mmol/L | 1.8 [1.4 - 2.2] | 1.8 [1.4 - 2.1] | 1.8 [1.3 - 2.3] | 0.645 |

*Abbreviations: NMBA: neuromuscular blocking agent; PEEP: positive end-expiratory pressure; PBW: predicted body weight; Crs: compliance of the respiratory system*

# FIGURES LEGENDS

**Figure E1: ROC curves for the ability of pH and PaO_2_ on FiO_2_ ratio to predict NMBA weaning failure**

*Abbreviations: ROC: receiver-operating characteristic; AUC: area under the ROC curve*

**Figure E2: ROC curve for the ability of mean tidal volume (mL/kg of PBW) during PSV to predict PSV failure**

*Abbreviations: ROC: receiver-operating characteristic; PBW: predicted body weight; PSV: pressure support ventilation; AUC: area under the ROC curve*

**Figure E3: ROC curve for the ability of mean tidal volume (mL/kg of PBW) during PSV to predict mortality at D28**

*Abbreviations: ROC: receiver-operating characteristic; PBW: predicted body weight; PSV: pressure support ventilation; AUC: area under the ROC curve*

**Figure E1**


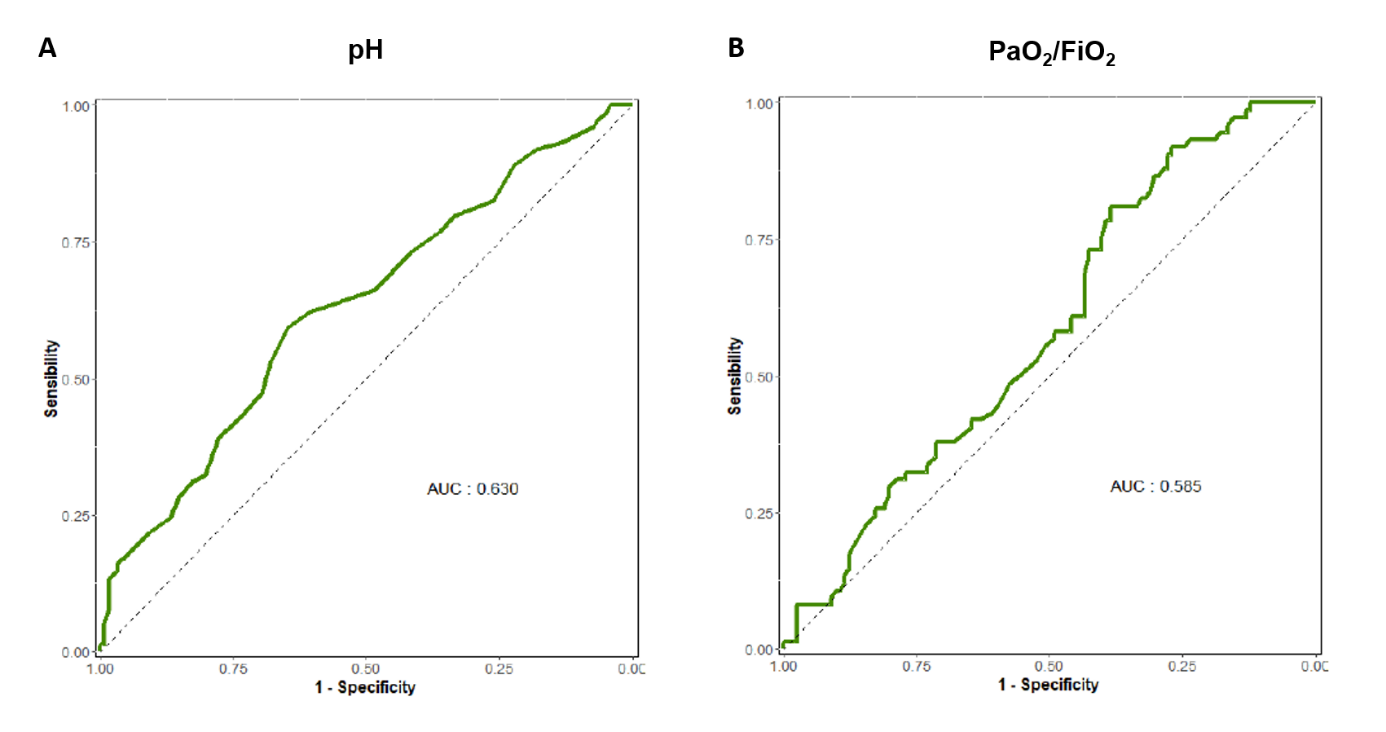


**Figure E2**


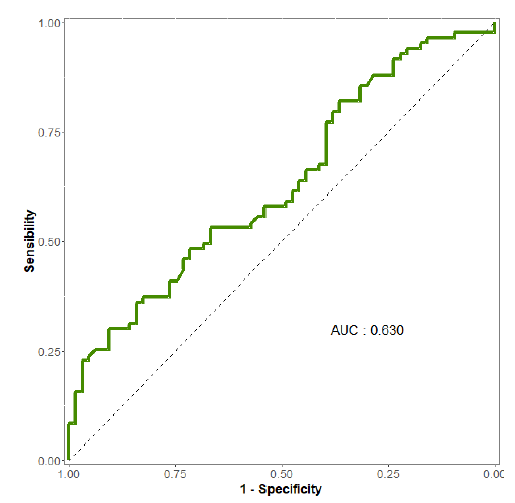


**Figure E3**


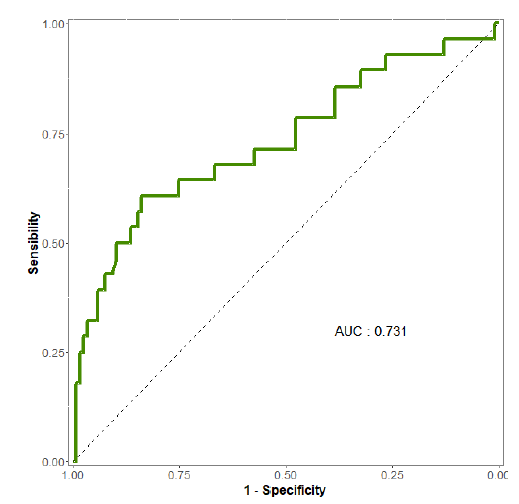

Supplement: Supplementary file 1 — Supplementary Material 1 [file 13613_2025_1484_MOESM1_ESM.docx]
